# Detection of SARS-associated Coronavirus in Throat Wash and Saliva in Early Diagnosis

**DOI:** 10.3201/eid1007.031113

**Published:** 2004-07

**Authors:** Wei-Kung Wang, Shey-Ying Chen, I-Jung Liu, Yee-Chun Chen, Hui-Ling Chen, Chao-Fu Yang, Pei-Jer Chen, Shiou-Hwei Yeh, Chuan-Liang Kao, Li-Min Huang, Po-Ren Hsueh, Jann-Tay Wang, Wang-Hwei Sheng, Chi-Tai Fang, Chien-Ching Hung, Szu-Min Hsieh, Chan-Ping Su, Wen-Chu Chiang, Jyh-Yuan Yang, Jih-Hui Lin, Szu-Chia Hsieh, Hsien-Ping Hu, Yu-Ping Chiang, Jin-Town Wang, Pan-Chyr Yang, Shan-Chwen Chang, members of the SARS Research Group of the National Taiwan University, National Taiwan University Hospital

**Affiliations:** * National Taiwan University, Taipei, Taiwan;; † National Taiwan University Hospital, Taipei, Taiwan;; ‡ National Health Research Institute, Taipei, Taiwan;; § Center for Disease Control, Taipei, Taiwan

**Keywords:** severe acute respiratory syndrome, SARS, coronavirus, CoV, Taiwan, perspective

## Abstract

The severe acute respiratory syndrome–associated coronavirus (SARS-CoV) is thought to be transmitted primarily through dispersal of droplets, but little is known about the load of SARS-CoV in oral droplets. We examined oral specimens, including throat wash and saliva, and found large amounts of SARS-CoV RNA in both throat wash (9.58 x 10^2^ to 5.93 x 10^6^ copies/mL) and saliva (7.08 x 10^3^ to 6.38 x 10^8^ copies/mL) from all specimens of 17 consecutive probable SARS case-patients, supporting the possibility of transmission through oral droplets. Immunofluorescence study showed replication of SARS-CoV in the cells derived from throat wash, demonstrating the possibility of developing a convenient antigen detection assay. This finding, with the high detection rate a median of 4 days after disease onset and before the development of lung lesions in four patients, suggests that throat wash and saliva should be included in sample collection guidelines for SARS diagnosis.

## Abstract 2

Early detection of SARS-CoV in throat wash and saliva suggests that these specimens are ideal for SARS diagnosis.

Severe acute respiratory syndrome (SARS) is an emerging infectious disease that spread rapidly from China to >30 countries, including Canada, Singapore, Vietnam, and Taiwan, in the first half of 2003 (*1**–**5*). In the latest update from the World Health Organization, the number of probable SARS cases is 8,096 (*5*). The etiologic agent of SARS has been identified as the novel SARS-associated coronavirus (SARS-CoV) (*6**–**9*). The disease is highly contagious and has the potential to cause a very large epidemic in the absence of control measures (*10**–**11*). Transmission appears to occur primarily through dispersal of droplets from the respiratory tract (*12*), generated when the patient talks, coughs, or sneezes (*4**,**5**,**10**–**13*). Although large amounts of SARS-CoV have been reported in sputum and nasal specimens, which may account for transmission during coughing and sneezing (*7**,**14*), little is known about the load of SARS-CoV in the oral cavity and how the virus is transmitted during talking. Since sneezing and rhinorrhea are not common symptoms of SARS, and cough with sputum is only seen in the later stage of infection (*1**–**3**,**13*), oral droplets generated during talking may represent an important route of transmission.

We examined specimens derived from the oropharynx and oral cavity, including throat wash and saliva, from 17 patients with probable SARS (*15**,**16*). Using a quantitative real-time reverse transcription–polymerase chain reaction (RT-PCR) assay and fractionation experiment, we investigated the load of SARS-CoV in these samples and different components of the throat wash.

## Materials and Methods

### Patients

From April 16, 2003, through May 1, 2003, during a 2-week period of the SARS outbreak in Taipei (*16*), 17 adult patients, who were admitted to the emergency department of the National Taiwan University Hospital and met the clinical case definitions for probable SARS (*15*), were included in this study. Physicians in the SARS Research Group of National Taiwan University Hospital made the diagnosis for each patient after thorough evaluation of their travel or contact history; symptoms; laboratory data including lymphopenia, thrombocytopenia, and elevated levels of lactate dehydrogenase or creatine kinase; and pneumonic patch in the chest x-ray. The first day of fever was defined as day 1 of illness. The serologic test of an indirect immunofluorescence assay performed on serum specimens collected 28 days after onset confirmed SARS-CoV infection in 13 of the 17 patients. The other four patients had at least two positive real-time RT-PCR results. Therefore, all 17 cases with probable SARS in this study were confirmed by laboratory tests (*15*).

### Sample Processing

With the patient's consent, saliva and throat wash (by gargling 10 mL normal saline) were collected in an airborne isolation room, according to the guidelines for aerosol-generating procedures (*17*). All samples were transferred to the biosafety level 3 (BSL3) laboratory and stored at –80°C until use (*18*). After thawing, 5 mL of the throat wash was centrifuged at 1,500 x *g* for 15 min to separate the supernatant from the mucous-cell pellet. Four milliliters of the supernatant were collected as the throat wash supernatant. The remaining 1-mL portion that contained the mucous-cell pellet was treated with equal volume of N-acetyl-L-cysteine at room temperature for 25 min and centrifuged at 1,500 x *g* for 15 min to further separate the cell pellet from the supernatant, of which 1.12 mL was collected as the treated supernatant of throat wash. Instead of extensively washing the potentially contagious cell pellet, we kept the remaining 0.88 mL as the cellular fraction of throat wash. Equal amounts of the supernatant, treated supernatant, and cellular fractions were subjected to viral RNA extraction. An aliquot of the saliva, to which an equal volume of 1 x phosphate-buffered saline (PBS) was added, was also subjected to viral RNA extraction.

### Isolation of Viral RNA

Viral RNA was isolated from aliquots of saliva and different fractions of throat wash from the 17 probable SARS patients and 12 healthy controls by using the QIAamp viral RNA mini kit (Qiagen, Hilden, Germany) in the BSL3 laboratory (*18*). Viral RNA was also isolated from culture supernatants of the SARS-CoV isolate, TW1 (*19*), human coronavirus 229E strain, and human enteric coronavirus Dallas 1 strain (American Type Culture Collection, Manassas, VA).

### Quantitative Real-Time RT-PCR

**Figure 1:**
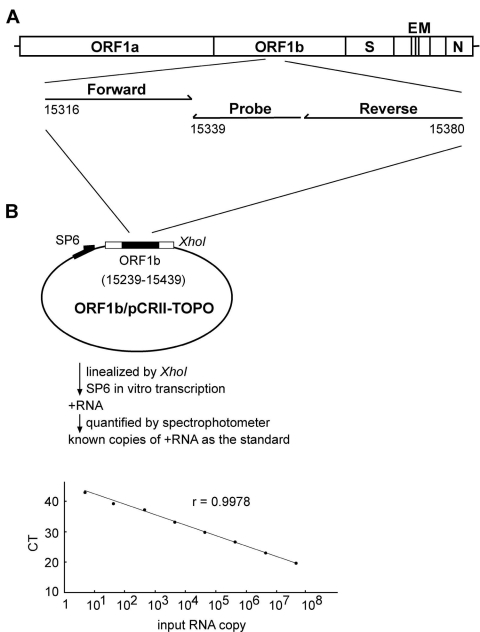
Quantification of the severe acute respiratory syndrome–associated coronavirus (SARS-CoV) RNA by real-time reverse transcription–polymerase chain reaction (RT-PCR) assay. (A) Location of the forward and reverse primers and probe in the genome of SARS-CoV, with the genome positions shown according to the Urbani strain (*20*). (B) A schematic diagram of the construct, ORF1b/pCRII-TOPO, and the protocol for generating the in vitro transcribed RNA as the standard for the real-time RT-PCR assay is shown. The relationship between known input RNA copies to the threshold cycle (CT) is shown at the bottom.

The assay used forward and reverse primers and a fluorogenic probe of the SAR1S_AS Taqman assay design (Applied Biosystems, Foster City, CA). They matched to a region within a previously described region of the ORF1b (*6**,**7*), which is also completely conserved by different isolates of SARS-CoV (Figure 1A) (20,21). The sequences of the forward primer, reverse primers, and probe are 5´-CACACCGTTTCTACAGGTTAGCT-3´ (genome positions 15316 to 15338 of the Urbani strain) (*20*), 5´-GCCACACATGACCATCTCACTTAAT-3´ ( positions 15380 to 15356) and 5´-ACGGTTGCGCACACTCGGT-3´ (positions 15355 to 15339), respectively. A 200-bp product covering this region was generated by using the primers (F1 and R1), the Superscript II one-step RT-PCR system (Invitrogen, San Diego, CA), and the RNA template derived from the SARS-CoV TW1 strain (*19*). The sequences of the primers F1 and R1 are 5´-CAGAGCCATGCCTAACATGC- 3´ (genome positions 15239 to 15258) (20) and 5´-GCATAAGCAGTTGTAGCATC-3´ (positions 15439 to 15420), respectively. RT-PCR conditions were 52°C for 40 min and 94°C for 2 min, followed by 35 cycles of 94°C for 1 min, 60°C for 1 min, and 68°C for 45 s. The product was subsequently cloned into the TA cloning vector (Invitrogen, San Diego, CA) to generate the construct, ORF1b/pCRII-TOPO (Figure 1B). The in vitro transcribed RNA was purified and quantified to determine the copy number of RNA as described previously (*22*). An aliquot (5 µL) of RNA isolated from the clinical sample and known amounts of the in vitro transcribed RNA (5 to 50 million copies) were subjected to real-time RT-PCR by using the SAR1S_AS primers, probe, and the Taqman one-step real-time RT-PCR master mix reagent kit (Applied Biosystems). The amplification conditions were 48°C for 30 min and 95°C for 10 min, followed by 45 cycles of 95°C for 15 s and 60°C for 1 min. The ABI prism 7000 sequence detector was used to analyze the emitted fluorescence during amplification. A positive result is defined by the cycle number (CT value) required to reach the threshold as described previously (*22*). Precautions for PCR were followed to avoid contamination (*23*). Since 5 µL of 50 µL RNA eluates that were derived from 560 µL throat wash supernatant, was used in each reaction, the number of SARS-CoV RNA copies per reaction was divided by 56 µL (560 µL x 5 µL/50 µL) and multiplied by 1,000 to determine the RNA copies per milliliter. The sensitivity of the assay is 5 copies RNA per reaction, corresponding to 90 copies per milliliter throat wash.

### SARS-CoV RNA in Components of Throat Wash

We used the following formula to calculate the copy numbers of SARS-CoV RNA in different components including the supernatant (S), the mucus-associated (M), and the cell-associated (C) components in the 5-mL throat wash, which was the starting volume in our fractionation experiment. The numbers of RNA copies in the S component equal the amount (copies/mL) in the supernatant times 5 (mL) (S = supernatant x 5 mL). Since treatment of the mucus-cell pellet with N-acetyl-L-cysteine presumably released SARS-CoV from the mucus and increased the volume twofold (from 1 mL to 2 mL), the copy numbers in the M component equal the amount in the treated supernatant (copies/mL) times 2 mL minus that from the originally untreated supernatant (1 mL) (M = treated supernatant x 2 mL – supernatant x 1 mL). The copy numbers in the C component equal the amount in the cellular fraction (copies/mL) times the volume of the cell pellet (C = cellular fraction x volume of cell pellet [in mL]). Taking patient ID17 as an example, S = 4,790 copies (958 x 5), M = 8,402 copies (4,680 x 2 – 958 x 1), and C = 330 copies (8,460 x 0.039). The amount of SARS-CoV RNA in the cell-free component, which equals the amount in the S component plus that in the M component, is 13,192 copies, corresponding to 97.5% of the total SARS-CoV RNA in 5 mL throat wash, and that in the cell-associated component is 330 copies, corresponding to 2.5% of the total (Table 1).

**Table 1:** Severe acute respiratory syndrome–associated coronavirus (SARS-CoV) RNA in cell-free and cell-associated components of throat wash from probable SARS cases

Patient ID^a^	Sampling day^b^	Copies/5 mL throat wash (% of total)	
		Cell-free component	Cell-associated component
1	d2	848,000 (99.6)	2,982 (0.4)
2	d3	34,320 (99.7)	85 (0.3)
3	d3	10,680 (99.7)	34 (0.3)
4	d3	119,500 (98.8)	1,495 (1.2)
5	d3	18,160 (99.6)	72 (0.4)
6	d4	14,400 (95.4)	695 (4.6)
7	d4	631,280 (99.9)	416 (0.1)
8	d4	19,900 (99.4)	124 (0.6)
9	d4	17,800 (99.5)	91 (0.5)
10	d5	40,500 (99.9)	60 (0.1)
11	d5	3,152,000 (99.2)	25,146 (0.8)
12	d6	1,896,000 (99.4)	12,160 (0.6)
13	d6	11,100 (99.6%)	40 (0.4)
14	d6	5,992 (99.4)	35 (0.6)
16	d8	33,400,000 (88.8)	4,222,000 (11.2)
17	d9	13,192 (97.5)	330 (2.5)

^a^Patients of probable SARS were diagnosed according to the World Health Organization clinical definitions (*15*). ^b^Sampling day 2 (d2) is the second day of fever.

### Immunofluorescence Assay

Aliquots of the cellular fraction of throat wash from patients and six healthy controls were fixed onto 12-well Teflon-coated slides and subjected to a previously described immunofluorescence assay (*19*). The first antibody was serum from a rabbit immunized with the recombinant nucleocapsid protein of the SARS-CoV (prepared by P.J. Chen), and the secondary antibody was the FITC-conjugated goat anti-rabbit immunoglobulin G (Pierce Biotechnology, Rockford, IL).

### Statistical Analysis

Regression analysis was performed to examine the correlation between the sampling day and the amount of SARS-CoV RNA in throat wash or saliva and the correlation between the amount in throat wash and saliva (software SPSS base 10.0, SPSS Inc., Chicago, IL).

## Results

The demographic and clinical information of the 17 patients are summarized in Table 2. Viral RNA was extracted from saliva and supernatant of the throat wash and then subjected to a quantitative real-time RT-PCR assay by using the primers and probe within a highly conserved region of the ORF1b (Figure 1A) (*20**,**21*). Known amounts of the in vitro transcribed RNA covering this region were used as the standard for quantification. As shown in Figure 1B, a linear curve was observed as the input RNA increased from 5 to 50 million copies per reaction. Positive signal was detected in the reactions containing RNA template derived from the SARS-CoV Taiwanese strain TW1 but not in those from 12 healthy controls and from two human coronaviruses (229E strain and human enteric coronavirus Dallas 1 strain) and not in the reaction containing no RNA (data not shown) (*19**,**22*).

**Table 2:** Clinical information and SARS-CoV RNA in throat wash and saliva from probable SARS case-patients^a^

Patient ID^b^	Age (y)	Sex	Sampling day^c^	Clinical information at sampling day					
				Fever	Cough	Dyspnea	Chest x-ray infiltrate	Throat wash (copies/mL)	Saliva (copies/mL)
1	52	M	d2	Yes	Yes	No^d^	Yes	1.58 x 10^5^	2.64 x 10^7^
2	28	F	d3	Yes	No	No	Yes	4.69 x 10^3^	1.12 x 10^5^
3	47	F	d3	Yes	No	No^d^	Yes	1.56 x 10^3^	1.06 x 10^5^
4	42	M	d3	Yes	No	No	Yes	2.39 x 10^4^	8.22 x 10^4^
5	26	M	d3	Yes	Yes	No	No	3.56 x 10^3^	1.22 x 10^4^
6	32	F	d4	Yes	No	No	Yes	2.88 x 10^3^	7.08 x 10^3^
7	48	M	d4	Yes	Yes	No	Yes	1.32 x 10^3^	9.05 x 10^4^
8	46	M	d4	Yes	No	No	No	3.98 x 10^3^	NA
9	41	F	d4	Yes	No	No^d^	No	3.56 x 10^3^	NA
10	47	M	d5	Yes	No	No	Yes	8.10 x 10^3^	9.24 x 10^4^
11	52	M	d5	Yes	No	Yes^d^	Yes	4.10 x 10^5^	1.74 x 10^7^
12	54	M	d6	Yes	Yes	No	Yes	2.46 x 10^5^	6.38 x 10^8^
13	48	F	d6	Yes	No	No	Yes	2.22 x 10^3^	1.78 x 10^5^
14	26	F	d6	Yes	No	No	Yes	9.73 x 10^2^	9.52 x 10^3^
15	21	F	d7	Yes	Yes	No	No	1.74 x 10^3^	NA
16	28	M	d8	Yes	No	No	Yes	5.93 x 10^6^	4.14 x 10^7^
17	25	F	d9	Yes	No	Yes	Yes	9.58 x 10^2^	2.80 x 10^4^

^a^SARS-CoV, severe acute respiratory syndrome–associated coronavirus; M, male; F, female; NA = not available. ^b^Probable SARS was diagnosed according to the World Health Organization clinical definitions (*15*). ^c^Sampling day 2 (d2) is the second day of fever. ^d^Intubation for hypoxia was performed during the course of hospitalization.

The results of the real-time RT-PCR assay on the throat wash and saliva are summarized in Table 2. The sampling day of these patients varied from day 2 to day 9 after onset of fever, with a median of day 4. SARS-CoV RNA was readily detected in throat wash from all 17 patients. The amount of the SARS-CoV RNA in the throat wash was 9.58 x 10^2^ to 5.93 x 10^6^ copies per mL (median 3.56 x 10^3^ copies/mL). SARS-CoV RNA was also detected in saliva from all 14 available specimens. The amount of SARS-CoV RNA in the saliva was 7.08 x 10^3^ to 6.38 x 10^8^ copies per mL (median 9.92 x 10^4^ copies/mL). The amount of SARS-CoV RNA in the throat wash or saliva does not correlate with the sampling day (simple linear regression, coefficient of correlation r = 0.106 and 0.147, respectively), underscoring a more complex course of virus-host interaction. The amount of SARS-CoV RNA in the saliva was greater than that in the throat wash for every patient from whom both type of specimens were available. A linear relationship existed between the amounts of SARS-CoV in the saliva and throat wash (simple linear regression, r = 0.848, p < 0.005), which suggests, but does not prove, that they could originate from a common source in the respiratory tract.

To further investigate whether SARS-CoV is also present in the cellular component of the throat wash, we carried out the fractionation experiment and examined the amount of SARS-CoV RNA in different components. As shown in Table 1, SARS-CoV RNA was detected in the cell-associated component of the throat wash from all 16 specimens examined. The range of viral load was 34–4222,000 copies per 5 mL throat wash. While 0.1%–11.2% of SARS-CoV RNA in the throat wash is present in the cell-associated component, a greater proportion of SARS-CoV RNA, 88.8% to 99.9%, is present in the cell-free component. This finding suggests that SARS-CoV is released very efficiently. The possibility that virus is released from the cells during thawing is unlikely, since the fractionation experiment performed for aliquots of some samples without prior freezing and thawing showed a similar result. For example, in ID7 the percentages of the cell-associated and cell-free components for an aliquot performed without freezing were 99.87% and 0.13%, respectively. These are similar to the results of 99.9% and 0.1% for another aliquot performed after freezing and thawing (Table 1).

**Figure 2:**
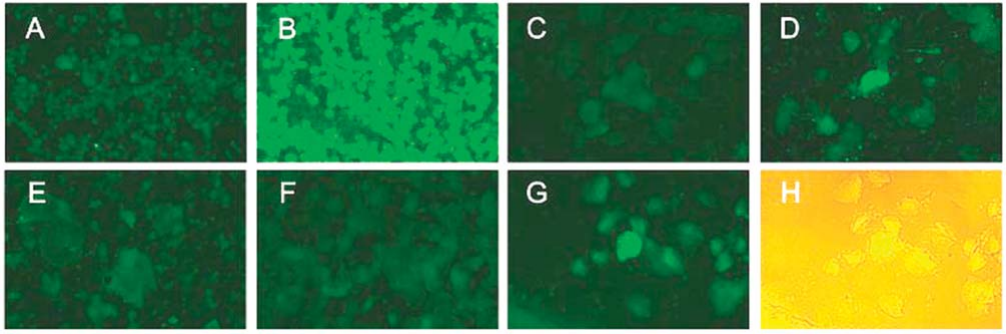
Detection of the severe acute respiratory syndrome–associated coronavirus (SARS-CoV) in the epithelial cells in throat wash from SARS patients by an indirect immunofluorescence assay. (A,B) Spot slides of SARS-CoV–infected Vero E6 cells were incubated with the preimmune (A) or postimmune (B) serum from a rabbit immunized with the recombinant nucleocapsid protein of the SARS-CoV, followed by fluorescein isothiocyanate–conjugated goat anti-rabbit immunoglobulin G. Panels A and B demonstrate the specificity of the reagents. (C to G) Epithelial cells in throat wash from a healthy control (E) and two SARS patients, ID17 (C,D) and ID11 (F,G), were incubated with the preimmune (C,F) or postimmune (D,E,G) rabbit serum. (H) The light microscopic picture of (G), taken with the fluorescent light on.

Electron microscopic studies have shown SARS-CoV particles in the desquamated cells from bronchoalveolar lavage and lung tissues, both in the lower respiratory tract (*6**,**8**,**24*). Our detection of SARS-CoV RNA in the cell-associated component of the throat wash suggested that SARS-CoV also replicates in the upper respiratory tract. To further explore this possibility, we prepared spot slides from the cellular fraction of the throat wash and examined them with an indirect immunofluorescence assay by using a polyclonal serum from a rabbit immunized with a recombinant nucleocapsid protein of SARS-CoV. When used in the epithelial cells prepared from two randomly chosen specimens (ID11 and ID17), the postimmune serum, but not the preimmune serum, reacted with epithelial cells with a speckle pattern (Figure 2, C–D and F–G). The classification of these cells as epithelial cells was supported by the size and morphologic features of them under light microscope (Figure 2H). Only background signal was seen in the cells prepared from a healthy control (Figure 2E). These findings indicate that SARS-CoV can replicate in the epithelial cells of the upper respiratory tract, and such cells can be used in an antigen detection assay.

## Discussion

We report large amounts of SARS-CoV RNA in the throat wash and saliva from probable SARS case-patients. This finding supports the possibility that SARS-CoV can be transmitted through oral droplets. Most coronaviruses are known to replicate in the epithelial cells of the respiratory or enteric tract. After budding into the pre-Golgi compartment, virus particles are released through an exocytosis-like process at the apical or basolateral surface, or both (*25*). Apical release is likely to facilitate the spread of virus in the respiratory or enteric tract, whereas basolateral release facilitates systemic spread. Our findings that SARS-CoV can be detected in cells derived from throat wash by the immunofluorescence assay and that most of the SARS-CoV in throat wash is present in the cell-free component suggest that after its replication in the epithelial cells, SARS-CoV is released efficiently and accumulates in the oropharynx and oral cavity, which may contribute to its transmission through oral droplets.

Practicing droplet and contact precautions prevents nosocomial transmission of SARS among healthcare workers (*26*). However, a cluster of SARS cases was reported among apparently protected healthcare workers during aerosol-generating procedures performed on SARS patients (*27*). This finding led to the controversial hypothesis of airborne transmission of SARS, in which very small particles (<5 µm) are spread in the air (*17**,**27*). A substantial proportion, 88.8 % to 99.9%, of the SARS-CoV in the throat wash was present in cell-free form; this finding offers a mechanistic explanation of the possibility of airborne transmission of SARS.

Three types of specimens from the upper respiratory tract, nasopharyngeal aspirates, nasopharyngeal swab, and oropharyngeal swab have been recommended to detect SARS-CoV (*28**,**29*). However, RT-PCR performed on nasopharyngeal aspirates from SARS patients had positive rates of 32% at day 3, 50% at day 5, and 68% at day 14 (*8**,**14*). A recent study using nasopharyngeal aspirates reported a positive rate of 71% at a mean of 4.4 days (*30*). In this study, we reported that SARS-CoV RNA can be detected in both throat wash and saliva from all specimens examined at an average sampling day 4.8 (range day 2–9). Furthermore, specimens of throat wash from four of our study participants who came to our emergency department, a designated SARS screening site in Taipei during the SARS outbreak, were collected when radiographic evidence of pneumonia or respiratory distress syndrome had not been observed (Table 2). To our knowledge, this report is the first showing that SARS-CoV could be detected in probable SARS patients before lung lesions developed.

The high SARS-CoV detection rate in our study contrasts with those reported previously by using nasopharyngeal aspirates (*8**,**14**,**30*). One possibility is that more SARS-CoV are present in the oropharynx and oral cavity than in the nasopharynx. In spite of the differences in the dilutional factors (for example, 10 mL of normal saline in the throat wash, 1.5–2 mL in the nasopharyngeal aspirates, and none in the saliva), the amounts of SARS-CoV RNA in the throat wash and saliva in our study, 9.58 x 10^2^ to 6.38 x 10^8^ copies per mL, were in the same range as those previously reported for the nasopharyngeal specimens (10^3^–10^8^ copies/mL) (*7**,**14*). A mutually nonexclusive possibility is that more respiratory secretions, mucus, and cells can be removed from the respiratory tract through throat wash than through nasopharyngeal aspiration, nasopharyngeal swabs, and oropharyngeal swabs. Regardless of the reason, SARS-CoV was also detected in the throat wash of nine SARS patients in a previous report (*6*), which suggests the need to evaluate the benefit of collecting throat wash to diagnose SARS.

To our knowledge, this report is the first that demonstrates the possibility of devising a SARS-CoV antigen detection assay by using cells derived from throat wash. Since a small number of patients were examined, future study with more patients and controls is required to develop a useful diagnostic test. Technically, throat wash and saliva are easier to collect when compared with the collection of currently recommended respiratory specimens (*28**,**29*). In addition, they can be obtained without close contact between the patient and healthcare worker, and thus reduce the risk for infection of healthcare workers. Another commonly obtained sample is sputum; however, it is rarely available at the early stage of infection, when virtually no cough or only dry cough is present (*1**–**3**,**13*). These features, together with the high detection rate at early stage and before the development of lung lesions, suggest that throat wash and saliva are ideal specimens for early diagnosis of SARS and should be included in guidelines for sample collection for SARS diagnosis (*28**,**29*). Further studies with longitudinally collected throat wash and saliva specimens from a larger number of SARS patients would help determine the onset and duration of infectiveness, extent of infectiveness of some patients, such as superspreaders, and the response to antiviral agents.
